# Revealing the extent of the first wave of the COVID-19 pandemic in Kenya based on serological and PCR-test data

**DOI:** 10.12688/wellcomeopenres.16748.1

**Published:** 2021-05-25

**Authors:** John Ojal, Samuel P. C. Brand, Vincent Were, Emelda A. Okiro, Ivy K. Kombe, Caroline Mburu, Rabia Aziza, Morris Ogero, Ambrose Agweyu, George M. Warimwe, Sophie Uyoga, Ifedayo M. O. Adetifa, J. Anthony G. Scott, Edward Otieno, Lynette I. Ochola-Oyier, Charles N. Agoti, Kadondi Kasera, Patrick Amoth, Mercy Mwangangi, Rashid Aman, Wangari Ng’ang’a, Benjamin Tsofa, Philip Bejon, Edwine Barasa, Matt J. Keeling, D. James Nokes

**Affiliations:** 1 Kenya Medical Research Institute - Wellcome Trust Research programme, Kilifi, Kenya; 2 Department of Infectious Disease Epidemiology, London School of Hygiene & Tropical Medicine, London, UK; 3 The Zeeman Institute for Systems Biology and Infectious Disease Epidemiology Research (SBIDER), University of Warwick, Coventry, UK; 4 School of Life Sciences, University of Warwick, Coventry, UK; 5 Health Economics Research Unit, Kenya Medical Research Institute - Wellcome Trust Research Programme, Nairobi, Kenya; 6 Population Health Unit, Kenya Medical Research Institute - Wellcome Trust Research programme, Nairobi, Kenya; 7 School of Public Health, Pwani University, Kilifi, Kenya; 8 Ministry of Health, Government of Kenya, Nairobi, Kenya; 9 Presidential Policy & Strategy Unit, The Presidency, Government of Kenya, Nairobi, Kenya; 10 Nuffield Department of Medicine, University of Oxford, Oxford, UK

**Keywords:** SARS-CoV-2, Kenya, dynamic model, serology, PCR cases

## Abstract

Policymakers in Africa need robust estimates of the current and future spread of SARS-CoV-2. We used national surveillance PCR test, serological survey and mobility data to develop and fit a county-specific transmission model for Kenya up to the end of September 2020, which encompasses the first wave of SARS-CoV-2 transmission in the country. We estimate that the first wave of the SARS-CoV-2 pandemic peaked before the end of July 2020 in the major urban counties, with 30-50% of residents infected. Our analysis suggests, first, that the reported low COVID-19 disease burden in Kenya cannot be explained solely by limited spread of the virus, and second, that a 30-50% attack rate was not sufficient to avoid a further wave of transmission.

## Introduction

The potential risk from severe acute respiratory syndrome coronavirus 2 (SARS-CoV-2) to Africa was identified early in the global pandemic ^1^. As the epicenter of transmission moved from East Asia to West Asia and Europe and then to North America, there was speculation as to the likely impact of the pandemic on the African continent with its young populations, high infectious disease burden, undernutrition and fragile health infrastructure. However, as health systems and economies of high-income countries strained, the reported burden of COVID-19 cases and associated deaths in Africa remained low with the exception of South Africa and Northern Africa ^2^. The question is whether this is the result of lower risk due to demographic structure (young age ^3^, either cross-reacting immunity (e.g. pre-existing SARS-CoV-2 cross-reactive T cells ^4^) or dampened immunological over-reaction ^5^, a low reproduction number from rapidly imposed interventions (such as school closures and lockdowns ^6^), environmental conditions (e.g. temperature and humidity ^7^), or under-reporting. The reason this remains a conundrum is, at least in part, a paucity of good quality data to reveal the probable extent of SARSCoV-2 spread in African populations.

Following the first confirmed coronavirus disease 2019 (COVID-19) case in Kenya on 13th March 2020, the Kenyan Government moved rapidly, closing international borders, schools, restaurants, bars and nightclubs, banning meetings and social gathering, and imposing a dusk to dawn curfew and movement restrictions in the two major city counties, Nairobi and Mombasa ^8^. The major concerns from unmitigated spread were a limited surge capacity of the Kenyan health system ^9^ and groups of the Kenyan population identified as potentially highly vulnerable to infection, due to socio-economic factors such as crowded households or lack of access to handwashing, and/or severe disease, due to epidemiological factors such as higher rates of obesity and hypertension ^10^. Throughout the months of April, May and into June 2020 few people in Kenya were reported SARS-CoV-2 test positive by polymerase chain reaction (PCR), or severely diseased or dying with COVID-19 as the established cause ^11^. There followed a relaxation of some measures in June and July including controlled opening of restaurants and places of worship and the removal of travel restrictions into and out of Mombasa and Nairobi counties. As of 30th September 2020, there were 45,795 laboratory-confirmed positive swab tests out of over 340,000 tests (about 13.5%), and 749 deaths with a positive test result in Kenya ^11^. This should be compared with the 200–250,000 cases and 30–40,000 deaths attributable to SARS-CoV-2 for similar sized countries in Europe (France, Italy, UK) by the end of September ^12^.

The reason for this apparently low level of COVID-19 disease in Kenya is unknown; one possible explanation is that SARS-CoV-2 had not widely spread among the Kenyan population by the end of September. However, two pieces of information suggest that SARS-CoV-2 had al-ready spread extensively by the end of September. First, a regionally-stratified seroprevalence study of 3098 Kenyan blood donors sampled between May and June reported a national estimate of 4.3% (adjusted to reflect the population distribution by age, sex and region) ^13^. Sero-prevalence was higher in Nairobi (7.6%) and Mombasa (8.3%). These levels of seropositivity are comparable to those reported in May in the United Kingdom (UK) ^14^, April/May in Spain ^15^, and March/April in some United States (US) cities ^16^, where high numbers of PCR-positive cases, hospitalizations and deaths have also been reported, in contrast to Kenya. Second, we noticed that test-positive PCR cases, and daily reported test-positive deaths, were declining in first Mombasa (from early July 2020) and then Nairobi (from early August 2020); respectively Kenya’s second and first largest cities. In Europe, declining case and mortality rates have been closely associated with non-pharmaceutical interventions (NPIs) ^17^. However, in Kenya this went counter to evidence of increased mixing, and hence reproduction potential, arising from Google Mobility data for these cities which showed a steady reversion in mobility towards pre-COVID-19 intervention levels since early April (Fig. S1). These observations, in turn, lead to the conclusion that either a smaller than expected proportion of infected individuals have had severe disease, and/or, that there has been under-reporting of severe disease.

To investigate these findings, we developed a simple SEIR (susceptible-exposed-infectious-recovered) compartmental mechanistic and data-driven transmission model for Kenya, which integrates three sources of longitudinal data: national time series polymerase chain reaction (PCR) tests, the Kenyan serological survey and Google mobility behavioural data. The overall modelling approach is similar to Flaxman *et al.* ^17^; that is we use time-to-event lag distributions, and the daily incidence time series, and, both models generate the daily incidence time series using a simple deterministic transmission model with the key unknowns being initial numbers of infected individuals and R(t). Where we differ in approach from Flaxman *et al.* ^17^ is that, instead of using reported test-positive deaths as the most reliable data for inferring underlying transmission patterns, we use a combination of PCR test-positive and serological data. The PCR test-positive data informs the model on the epidemic trajectory but does not account for likely under-detection of cases. This under-detection of cases is inferred from the proportion exposed to SARS-CoV-2 evidenced by the seroprevalence estimates, hence scaling the incidence estimation. Finally, the mobility data, as a proxy for the contact rate, determines the contribution of the intervention (which acts to alter contact patterns) relative to other factors that alter incidence and the effective reproduction number, the most important of which is the susceptible proportion of the population. Our aim is to derive a coherent picture of the SARS-COV-2 epidemiology in Kenya in the first wave and reveal the historic and future patterns of spread across the country and by county. Reported deaths are not used as primary data for inference, but rather the trend in changing rates of reported deaths is used as a validation data set for model predictive accuracy (see supporting information for description of model validation). Reported deaths may be subject to substantial under-reporting, and we assume that the bias in under-reporting is consistent over time.

## Results

### Underlying transmission rates in Mombasa and Nairobi during the first wave

**Figure 1.**
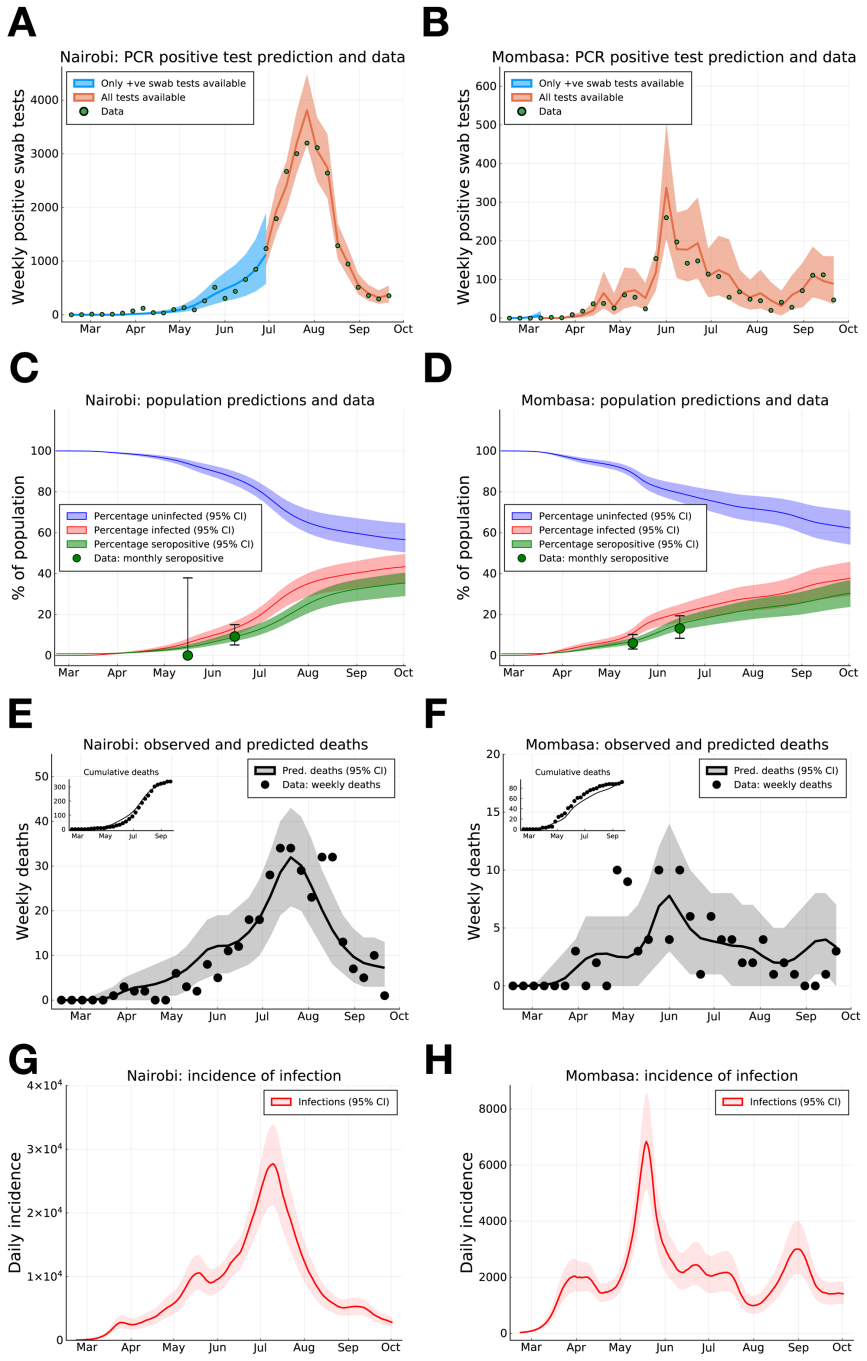
SARS-CoV-2 PCR positive swab tests, seroprevalence and deaths in Nairobi and Mombasa, Kenya, with model forecasting. ( **A**) and ( **B**) Weekly reported positive PCR positive swab tests (green dots) for Nairobi ( **A**) and Mombasa ( **B**), model prediction of mean weekly detection during both sampling periods when negative PCR test data was unavailable (blue curve), and available (orange curve). ( **C**) and ( **D**) Monthly seropositivity of Kenya National Blood Transfusion Service (KNBTS) blood donors in Nairobi ( **C**) and Mombasa ( **D**) (green dots), model predictions for population percentage of seropositivity (green curve), exposure to SARS-CoV-2 (red curve), and uninfected (blue curve). ( **E**) and ( **F**) Daily deaths with a positive SARS-CoV-2 test in Nairobi ( **E**) and Mombasa ( **F**) by date of death (black dots), and model prediction for daily deaths (black curve). Inset plots in ( **E**) and ( **F**) indicate cumulative reported deaths and model prediction. ( **G**) and ( **H**) Model estimates for rate of new infections per day in Nairobi ( **G**) and Mombasa ( **H**). Background shading indicates 95% central credible intervals. Dates for all graphs mark the 1st of each month.

**Figure 2.**
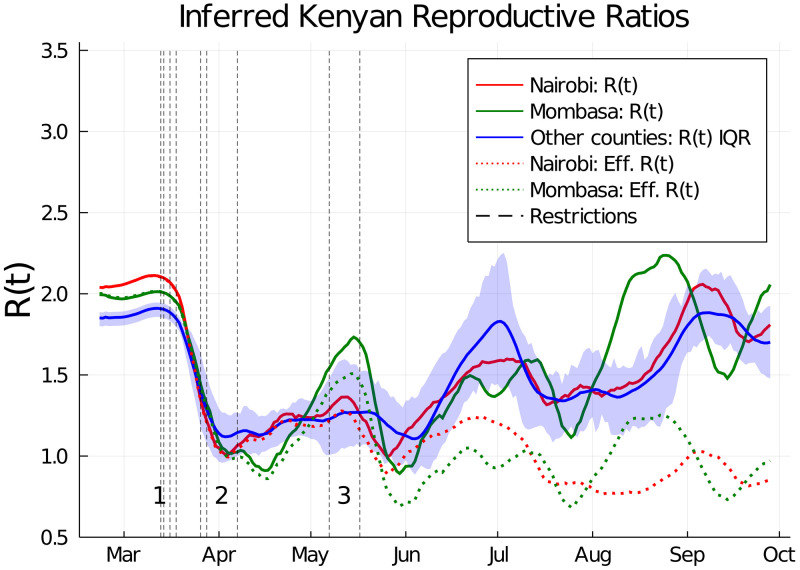
Estimated basic and effective reproductive numbers in Kenya since Feb 21st 2020. The posterior mean reproductive number for Nairobi (red curves), Mombasa (green curves), and the inter-quartile range (IQR) over mean reproductive number estimates for all other Kenyan counties (blue curve and shading). Shown are both the basic reproductive numbers (expected secondary infections in a susceptible population adjusted for mobility changes since the epidemic start; solid curves), and effective reproductive numbers (expected secondary infections accounting for depletion of susceptible prevalence in the population; dotted curves). The effective reproductive number varied significantly from county to county and is not shown except for Mombasa and Nairobi. Restrictions aimed at reducing mobility in risky transmission settings (black dotted lines) are labelled in groups. The chronologically ordered restrictions in each group are: 1) First PCR-confirmed case in Kenya, suspension of all public gatherings, closure of all schools and universities, and retroactive quarantine measures for recent returnees from foreign travel, 2) suspension of all inbound flights for foreign nationals, imposition of a national curfew, and regional lockdowns of Kilifi, Kwale, Mombasa and Nairobi counties, and 3) additional no-movement restriction of worst affected areas within Mombasa and Nairobi, and, closure of the border with Somalia and Tanzania.

As at 30th September, a substantial proportion of PCR positive tests have been samples from the capital Nairobi (25,182 positive tests), while Kenya’s second largest city, Mombasa, has reported the next highest number of PCR positive tests (2,056). We infer that the underlying rate of new infections peaked on May 18th 2020 (CI May 16th - May 21st) in Mombasa and July 9th 2020 (CI July 7th - July 10th) in Nairobi, and subsequently declined from peak transmission ( Figure 1 H, G). The model suggests that the PCR test and serology data can be explained by the initial presence of <200 infected individuals in both Mombasa and Nairobi on 21st February, three weeks before the first reported case in Kenya. Thereafter, growth of transmission was rapid in both counties. In early March, the reproductive ratio was estimated to be 1.94 (CI 1.89-1.98) and 2.00 (CI 1.97-2.02) in Mombasa and Nairobi, respectively, with associated doubling-time of 4.84 and 4.59 days, respectively. After March, the transmission curves flattened substantially. This change is consistent with the introduction of containment measures by the Kenyan government, and evidence of substantial reduction in mobility (see Google Mobility data Fig. S1). From late April, through May and June, and into July the evidence suggests movement restrictions became steadily less effective. The waning effectiveness of movement restrictions results in an inferred increase in R(t) across Kenyan counties and an increased rate of epidemic growth ( Figure 2). The increasing R(t) estimates are broadly in line with predicted trends from Google mobility data (supporting information), although it should be noted that the R(t) estimates exhibit secondary fluctuations around the increasing mobility trend ( Figure 2). In Nairobi and Mombasa we predict that reduction in susceptibility of the population ( Figure 1C,D) caused the effective reproductive ratio (R _*eff*_; the mean number of secondary cases accounting for reduced susceptibility) to drop significantly below the basic R value from June onwards ( Figure 2). However, other counties, where the epidemic did not establish itself as early as Mombasa and Nairobi, and where a substantial majority of the population are likely to still be susceptible, now have R(t) estimates which we estimate rebounded to the original levels estimated as occurring before Kenyan public health measures ( Figure 2).

By accounting for the delay of an average of 19 days between infection and death (supporting information for details on infection to death distribution) we find the transmission curve, estimated from PCR tests and serology, generates a good prediction of the observed trend in daily deaths in Nairobi and Mombasa ( Figure 1 E, F). We did not use mortality data in transmission model inference, therefore the good fit to the observed trend in deaths with a PCR-confirmed test result represents an out-of-sample validation of the modelling ^18^. Note, it is the distribution of deaths over time, rather than the absolute numbers, that we consider to be a good fit. In accord with observations, we estimate a peak of positive PCR test samples occurred at the end of July or early August in Nairobi and earlier, mid-June, in Mombasa. The lag between transmission peak and positive swab testing peak being explained by both the delay between infection and becoming detectable by PCR, and the period after an infected individual has ceased being actively infectious but remains detectable by PCR ^19^ ( Figure 1 G,H and A,B). As of the end of September 2020 we estimate that about 35.4% (CI 29.0%-40.4%) of the Nairobi population, and 30.3% (CI 23.6-36.7%) of the Mombasa population were serologically positive with SARS-CoV-2, ( Figure 1 C,D). This estimated level of seropositivity is substantially higher than has been estimated in some countries that have been hit hard by the pandemic ^14– 16^. However, they are in broad agreement with a study in Niger state, Nigeria, from June 2020 ^20^, as well as seropositivity rates reported from the hard-hit city of Manaus, Brazil, in May 2020 ^21^. Note that these estimates of seropositivity at the end of September assume both that waning seropositivity would not have had a significant effect on serological observations by late September, and furthermore that waning immunity leading to re-infection remained insignificant by late September.

### SARS-CoV-2 attack rates in the first wave in Kenyan counties and the estimated crude infection-to-fatality ratio

Accounting for the sensitivity of the serological assay, and the delay between infection and seroconversion, we estimate that the actual exposure of the population to SARS-CoV-2 by September 30th was 43.3% (CI 35.3%-49.5%) in Nairobi and 37.6% (CI 29.2%-45.7%) in Mombasa ( Figure 1 C,D). Such levels of population exposure are predicted to be associated with decreased rates of new cases due to reduced numbers of susceptible individuals in these urban populations, although this is also influenced by the estimated reproductive number and effective population size at risk of exposure ( *P _eff_*). The effective population size accounts for the impact of heterogeneity in the susceptibility, transmissibility and social interactivity in the population (supporting information for more details on effective population size in transmission modelling); for Nairobi it was inferred as 81.8% of actual population size (CI 66.7%-93.2%), for Mombasa 71.9% (CI 56.3%-86.5%). The effective population size estimates rest upon inferred variation in risk across the population. There remains a possibility of future increase in transmission if population mobility continues to rise, if population mixing patterns alter leading to changed risk heterogeneity or if immunity is short lived, leading to a rebound in reported cases. One or more of these factors could lead either to lengthening the tail after the first peak in cases/deaths, or even to a secondary increase in cases and/or deaths.

The inferred *IFR _crude_* values for both Nairobi ( *IFR _crude_* = 0.019% (CI 0.014%-0.024%) and Mombasa ( *IFR _crude_* = 0.022% (CI 0.016%-0.027%)) are substantially lower than the age-adjusted IFR expected for Kenya under full ascertainment from the age-specific IFR estimated given by Verity *et al.* ( *IFR _verity_* = 0.26% ^22^; and supporting information). This is a crude observational value for the infection to fatality ratio, since we do not currently have an estimate of the reporting bias of deaths of individuals infected with SARS-CoV-2. Therefore, our estimate of *IFR _crude_* potentially reflects lower detection in Kenya compared to China, as well as any lower mortality risk due to fewer comorbidities.

**Figure 3.**
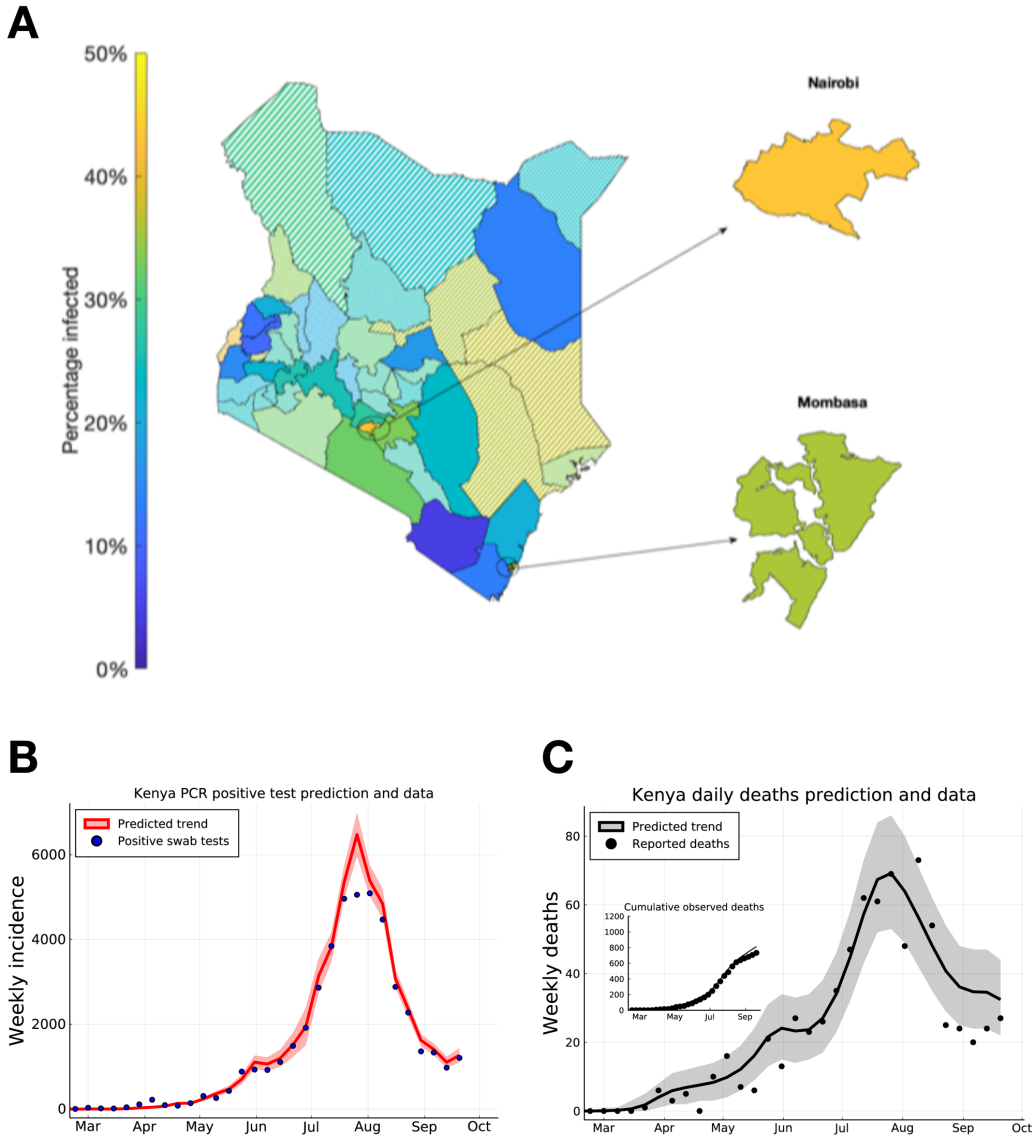
Predicting peak timing of transmission rate by Kenyan county, and forecasting of Kenya-wide PCR positive swab tests and reported deaths. ( **A**) Posterior mean estimates for the attack rate (% of population) in each county. Solid shaded counties have a posterior standard deviation in their attack rate estimate of less than 10%, candy-stripe shaded counties have greater uncertainty associated with their attack rate estimate. ( **B**) Kenya total positive swab tests collected by day of sample (blue dots) with model prediction of daily positive swab test trend (red curve). ( **C**) Kenya total reported deaths with a positive swab test (black dots), with model prediction of reported death rates (black curve). Inset plot indicates cumulative reported deaths with model prediction of cumulative deaths. Dates on ( **B**) and ( **C**) mark 1st of the month.

We extended our model-based inference to each of the 47 counties in Kenya (see dataset S1 for parameter estimates, peak time estimates and *IFR _crude_* estimates for each county). We find that, in addition to the two main Kenyan city counties, more than 25–30% of the population in each of the semi-urban counties neighbouring Nairobi (Kiambu, Kajiado, and Machakos) had been infected. However, the infection rate is predicted to be either lower than 25% and/or subject to high uncertainty in other counties (with high uncertainty defined as a prediction standard error of > 10% of county population size; Figure 3).

Due to the lag between infection and the observability of the infected person (whether by swab PCR test, serology test, or death), we estimate that both daily PCR positive test detections and daily observed deaths attributed to COVID-19 across the two main cities, and semi-urban counties neighbouring Nairobi had a peak in early August 2020 ( Figure 3 B,C). Hospitalisation rates are not available for all Kenyan hospitals. However, sentinel clinical surveillance of severe acute respiratory infection (SARI), with or without a PCR test for SARS-CoV-2, at 14 county hospitals suggests an increasing rate of adult admissions in June and July 2020 ^23^. However, SARI admissions were lower in the early phase of the Kenyan epidemic than observed counts from the same months in 2018 and 2019 ^23^ and the apparent rise in SARI admissions could represent a reversion towards pre-COVID numbers; this observation underlines the difficulties in using hospital data to understand the penetration of SARS-CoV-2 in Kenya.

## Conclusions and Discussion

Our modelling analysis provides a coherent account of the SARS-CoV-2 pandemic in Kenya up to end September 2020. Limitations include lacking information on the PCR testing denominators for the full time frame, the limited serological survey and that we have applied a simple dynamic model. In mitigation similar results were obtained when excluding all negative tests, and the dynamic model is transparently a fit to the data where the availability of the latter is a key strength of our study.

Our analysis suggests that 30–50% of the urban population were already exposed by the end of September, and that the first wave of the Kenya epidemic peaked in the urban and semi-urban counties during a period of relatively little restrictions or physical distancing. This infers a burden of infection in Nairobi and Mombasa similar to extremely hard-hit cities in South America at the same time, e.g. Manaus ^21^. However, in Manaus there was a substantial first epidemic wave through April-June 2020, with about 50% of the population infected, followed by a long epidemic tail leading to an estimated total 75% attack rate by the end of 2020 ^21^. In Kenya, the second wave came shortly after the first (October to December 2020), which suggests that the causes of the Kenyan second wave are complex.

Whilst the full picture of the epidemiology in Kenya will not be established until cause-specific mortality data become available (e.g. from resumption of Demographic Surveillance System and verbal autopsy activities), our model, fitted to three sources of nationwide longitudinal data, suggests that the number of symptomatic COVID-19 cases reported and the mortality attributed to the SARS-CoV-2 epidemic are substantially lower in Kenya than in Europe and the USA at a similar stage of the epidemic. This would remain the case even if reported deaths accounted for just 1/10th of the true value. However, there is insufficient data for speculating on the degree of under-reporting and previous estimates of 1 in 4 deaths occurring in hospital may not be generalizable to the hospital access during the COVID-19 pandemic ^24^.

Late 2020 saw the spread of COVID-19 to more rural areas of Kenya, with less infrastructure and access to public health facilities and a second wave of SARS-CoV-2. This second wave needs to be dissected and understood. Policy makers need to balance the direct and indirect health and socio-economic consequences of any control measures; a balance that becomes more precise as we develop a better understanding of SARS-CoV-2 dynamics in Kenya.

## Methods

### Transmission model definition

The dynamics of transmission in each Kenyan county were assumed to follow a SEIR transmission model with an effective population size parameter ( *P _eff_*) ^25^. The SEIR model with effective population size is an extension of the homogeneous SEIR model ^26^ with the additional flexibility that *P _ef f _N* out of a total population size *N* in each county is at risk of contracting SARS-CoV-2. *P _eff_* =1 recovers the homogeneous SEIR model, whereas, *P _ef f _<*1, recovers the effect of underlying heterogeneity in the transmission potential and risk in the population of the county on the aggregate dynamics of epidemic. This aspect of heterogeneous models of transmission has been widely investigated, for example, in the context of comparing vaccination coverage thresholds for elimination between uniform and targeted vaccination policies ^27^. In the context of the SARS-CoV-2 pandemic modelling literature, the possible role of population heterogeneity in decoupling estimates of *R*_0 _from predictions of the "herd-immunity" threshold and final attack rate has again been identified ^28, 29^. In this study, rather than make strong assumptions about the mechanism of population heterogeneity, e.g. differential susceptibility, differential rates of social mobility etc., we have taken a phenomenological approach; the effect of heterogeneity in the population was encoded in the effective population parameter *P _eff_*, and this parameter was inferred jointly with *R*_0_. Our *a priori* belief was that the most probable value was *P _eff_* = 1. We assumed that *P _eff_* was constant over the period of inference.

The model dynamics for each Kenyan county were represented as a system of ordinary differential equations,

$$\;\begin{array}{r} \;\dot{S}(t)=-\gamma R_{t}\frac{S(t)I(t)}{P_{eff}N},\\ \dot{E}(t)=\gamma R_{t}\frac{S(t)I(t)}{P_{eff}N}-\sigma E,\\ \dot{I}(t)=\sigma E(t)-\gamma I(t),\\ \dot{R}(t)=\gamma I(t),\\ \dot{C}(t)=\gamma R_{t}\frac{S(t)I(t)}{P_{eff}N}. \end{array}\;(1)$$

With initial conditions (time 0 is the calendar date 21st Feb 2020 and all rates are per day),

$$\begin{array}{l} S(0)=P_{eff}N-E_{0}-I_{0},\\ \;E(0)=E_{0},\;I(0)=I_{0},\;R(0)=0,\;C(0)=0. \end{array}\;(2)$$

Where the dynamic variables *S*( *t*), *E*( *t*), *I*( *t*), *R*( *t*) were the numbers of susceptibles-at-risk, exposed (but not yet infectious), infectious, and, recovered individuals in the county. The full number of susceptibles in the county at any time was (1 − *P _eff_*) *N*+ *S*( *t*). *C*( *t*) was the cumulative numbers of infected individuals in the county.

The incubation-to-infectious rate was *σ* = 1 */*3.1 per day, and the recovery rate was *γ* = 1 */*2.4 per day, implying a mean generation time of 5.5 days (see *Supporting information* for a comparison to the generation distribution inferred by Ferretti *et al.* ^30^). The instantaneous reproductive ratio *R*_t_ *= R*_0_ *β*_t _decomposed into a basic reproductive ratio *R*_0 _and an effective contact rate *β*_t _, where *β*_t _= 1 represents a pre-pandemic baseline contact rate in the population.

### Transmission model inference

We used a mixed Bayesian and maximum a-posteriori (MAP) approach to parameter inference for each of the 47 Kenyan counties, based on daily observations of positive and negative PCR and serology tests in each county. The likelihood of individuals being detectable on any given day was based on whether they had been infected before that day, and, the number of days since their infection. The number of new infections on each day *n*, was denoted *ι _n_*. For a given set of model parameters *ι*_n _was generated by solving the ODE system ( 1), giving,

$$\;\iota_{n}=C(n+1)-C(n),\;(3)$$

for each day *n*. Given the daily numbers of new infections, the number of people in the county on each day *n* who are detectable by PCR testing, denoted ( *P* ^+^) _*n*_, and serological testing, ( *S* ^+^) _*n*_, were given by convolving the new infection time series with the probability of (respectively) being detectable by a PCR or serological test *τ* days after infection, *Q _PCR_* ( *τ*) and *Q _sero_* ( *τ*):

$$\;\begin{array}{l} {\left(P^{+}\right)}_{n}=[\iota *Q_{PCR}](n),\\ {\left(S^{+}\right)}_{n}=[\iota *Q_{sero}](n). \end{array}\;(4)$$

The log-likelihood function for each county has the form,

$$\;\begin{array}{l} l({\left(P^{+}\right)}_{1},...,{\left(P^{+}\right)}_{T},{\left(S^{+}\right)}_{1},...,{\left(S^{+}\right)}_{T},\;\theta_{OM})=\\ \;\sum_{n=1}^{T}\ln\;f_{PCR}({\left(ObsP^{+}\right)}_{n}|{\left(P^{+}\right)}_{n},\theta_{OM})\\ \;+\ln\;f_{sero}({\left(ObsS^{+}\right)}_{n}|{\left(S^{+}\right)}_{n},\theta_{OM}). \end{array}\;(5)$$

Where, ln *f _PCR_* (( *ObsP* ^+^) _*n*_|( *P* ^+^) _*n*_, *θ _OM_*), and, ln *f _sero_* (( *ObsS* ^+^) _*n*_|( *S* ^+^) _*n*_, *θ _OM_*), were, respectively, the log-probability of observing ( *ObsP* ^+^) *_n_* PCR test-positives and ( *ObsS* ^+^) _n _serological test positives on days *n* = 1,..., *T* given the model prediction of numbers of PCR and serological detectable people in the population, and the observation model parameters *θ _OM_*. Day *n* = 1 corresponded to the calendar date 21st Feburary 2020, and, day *n* = *T* = 223 corresponded to 30th September 2020. The observation model parameters included bias and over-dispersion parameters for finding PCR positives in the daily testing group compared to an unbiased binomial sample over the population; we used a beta-binomial count data model whenever the number of negative PCR tests for that day was available in the county and a negative binomial count data model whenever it was not. We assumed that the serological test-positives were from an unbiased sample but included over-dispersion in sampling serological positives by using a beta-binomial count data model. *Supporting information* gives further details on the data sources and the log-likelihood calculation including a full description of all observation model parameters and the functional forms and underlying evidence for *Q _PCR_* and *Q _sero_*. The data sources used were: The Kenya Ministry of Health National linelist, the Kenya Medical Research Institute Wellcome Trust Research Programme (KEMRI-WTRP) serological surveillance programme and Google mobility data ^31^. The full Kenyan SARS-CoV-2 line list contains sensitive personal information that could potentially allow the identification of individual cases. The analysis performed in this study only required an aggregated dataset derived from the Kenyan linelist. Other data used in this paper was openly available. All data is available in the main text or as underlying data ^32^.

We assumed that *β*_t _was piece-wise constant on days, and, therefore, could be reconstructed from daily effective contact rates ( *β _n_*) _*n*=1,..., *T*_. For any fixed estimate of the effective contact rate *β*_t _, we used Hamiltonian Markov-chain Monte Carlo (HMC) ^33^ to estimate the posterior distribution for the transmission model parameters; that is the initial condition values ( *E*_0_, *I*_0_) and fixed parameters ( *P _eff_*, *R*_0_) jointly with the observation model parameters *θ _OM_*. Prior distributions for parameters were chosen for groups of counties (e.g. largely rural counties had different priors to major urban conurbations like Nairobi and Mombasa; see *supporting information* for further details). Starting from an initial estimate that *β _t_* followed daily Google mobility trends ^31^ for the whole period, we sequentially improved our *β _t_* estimate using the expectation-maximisation (EM) algorithm ^34^. The E-step corresponding to posterior distribution estimation using HMC, and the M-step corresponding to optimising the daily effective contact rate estimates ( *β _n_*) _*n*=41,..., *T*_ using the popular stochastic gradient descent algorithm ADAM ^35^. The first 40 days of effective contact rate estimates ( *β _n_*) _*n*=1,...,40 _were assumed to be fixed to their Google estimate; this improved identifiability jointly with *R*_0 _and captured the observed sharp drop in mobility in response to Kenyan public health measures following the first identified case on 13th March 2020. See *supporting information* for further details on the use of Google mobility data and the EM algorithm method used in this study.

After inference of transmission parameters, the model implied a prediction of the expected number of daily deaths due to COVID, ( *X* ^+^) _*n*_, based on an overall population infection-to-fatality ratio (IFR), and, the delay distribution between infection and death, *p _ID_*,

$$\;E{\left(X^{+}\right)}_{n}=IFR[\iota *p_{ID}](n).\;(6)$$

In this study, we assume that the IFR is constant for each county over the period of inference, which allows us to construct a Bayesian estimator of the crude IFR, *IFR _crude_*, by fitting to the observed daily numbers of test-positive deaths, ( *ObsX* ^+^) _n _(see *supporting information* for details and background data informing *p _ID_*). Because the observed test-positive deaths were not used in inferring model parameters, we treat the log-predictive density of deaths from the model as an out-of-sample validation metric for the model. However, we emphasise that the out-of-sample comparison is to the **trend** of daily deaths, because this is invariant to the *IFR _crude_* estimator, which is itself sensitive to under-reporting of COVID deaths. *Supporting information* gives full details on the Bayesian model validation used in this study.

This study was approved by the Kenya Medical Research Institute Scientific and Ethics Review Unit (KEMRI-SERU) with approval numbers KEMRI/SERU/CGMR-C/203/4085 and KEMRI/SERU/CGMR-C/203/3426 for the modelling and serosurvey studies respectively.

## Data availability

### Underlying data

Zenodo: Revealing the extent of the first wave of the COVID-19 pandemic in Kenya based on serological and PCR-test data. https://doi.org/10.5281/zenodo.4705244 ^32^ This project contains the following underlying data:

- Data S4 (The number of positive, and negative where available, PCR-confirmed swab tests for each county by date of sample collection (21st Feb to 30th September)).
- Data S5. (The number of positive and negative sero-logical results for each county by date of sample collection (21st Feb to 6th August)). This is from the **Kenyan Ministry of Health National linelist**.
- Data S6. (The number of deaths with a PCR-confirmed swab test for each county by recorded date of death (21st Feb to 30th September)).
- Data S7. (Summary data of Kenyan epidemic, including reported total number of test performed in Kenya.
- supp material.docx (A more detailed description of the data)

## Software availability

The analysis code was written in Julia language version 1.4.

- The code base underlying the analysis is accessible at the open github repository https://github.com/ojal/KenyaSerology. For the analysis presented here we developed a module in the Julia programming language called *KenyaSerology*. Tutorial notebooks for running *KenyaSerology* and analysing the underlying data are available in the repository.
- Archived source code at time of publication: https://doi.org/10.5281/zenodo.4705244 ^32^
- License: GNU General Public License v3.0
