# Supplementary material for: Revealing the extent of the first wave of the COVID-19 pandemic in Kenya based on serological and PCR-test data
Source: Wellcome Open Res. 2022 Sep 22;6:127. Originally published 2021 May 25. [Version 3] doi: 10.12688/wellcomeopenres.16748.3 (PMC9511207; doi:10.12688/wellcomeopenres.16748.3)
Supplement: Peer review history

# Peer review: Reviewer response for version 3

**DOI:** 10.21956/wellcomeopenres.20370.r52507

**Published:** 2022-09-23

**Authors:** Benjamin J. Cowling

**Affiliations:** 1 WHO Collaborating Centre for Infectious Disease Epidemiology and Control, School of Public Health, The University of Hong Kong, Hong Kong, Hong Kong

No further comments.

Is the work clearly and accurately presented and does it cite the current literature?

Yes

If applicable, is the statistical analysis and its interpretation appropriate?

Yes

Are all the source data underlying the results available to ensure full reproducibility?

Yes

Is the study design appropriate and is the work technically sound?

Yes

Are the conclusions drawn adequately supported by the results?

Yes

Are sufficient details of methods and analysis provided to allow replication by others?

Yes

Reviewer Expertise:

Infectious disease epidemiology

I confirm that I have read this submission and believe that I have an appropriate level of expertise to confirm that it is of an acceptable scientific standard.

# Peer review: Reviewer response for version 2

**DOI:** 10.21956/wellcomeopenres.19588.r51227

**Published:** 2022-06-27

**Authors:** Benjamin J. Cowling

**Affiliations:** 1 WHO Collaborating Centre for Infectious Disease Epidemiology and Control, School of Public Health, The University of Hong Kong, Hong Kong, Hong Kong

This is a nice study. The authors used national surveillance PCR test data, serological data and mobility data to develop and fit a county-specific transmission model for Kenya up to the end of September 2020, which encompasses the first wave of SARS-CoV-2 transmission in the country. Authors estimated that the first wave of the SARS-CoV-2 pandemic peaked before the end of July 2020 in the major urban counties, with 30-50% of residents infected.

 This is an important study and likely has implications for other neighbouring countries in Africa as well. Data on COVID-19 from the African continent are very limited. I encourage indexing of this revised submission and I just had two minor comments:

- Abstract final phrase - "further wave of transmission" do you mean specifically a further wave of transmission with an antigenically-different strain such as a new variant which can escape the population immunity that has built up? Or you mean that the first wave was controlled before population immunity reached a herd immunity threshold? Even so, if the same strain circulates again, one wouldn't expect a large wave because of the existing immunity from the first wave. Waning immunity in medium-term could also play a role in allowing subsequent epidemics.
- Conclusions - I don't find the Manaus estimate of 75% particularly compelling due to methodological issues in that study. There should be other locations with less extreme first-year serological data?

Is the work clearly and accurately presented and does it cite the current literature?

Yes

If applicable, is the statistical analysis and its interpretation appropriate?

Yes

Are all the source data underlying the results available to ensure full reproducibility?

Yes

Is the study design appropriate and is the work technically sound?

Yes

Are the conclusions drawn adequately supported by the results?

Yes

Are sufficient details of methods and analysis provided to allow replication by others?

Yes

Reviewer Expertise:

Infectious disease epidemiology

I confirm that I have read this submission and believe that I have an appropriate level of expertise to confirm that it is of an acceptable scientific standard.

# Response

**Published:** 2022-09-05

**Authors:** James Nokes

**Affiliations:** KEMRI-Wellcome Trust Reseach Programme, Kenya

We thank the reviewer for useful review and comments.

*Point 1 “Abstract final phrase - "further wave of transmission" do you mean specifically a further wave of transmission with an antigenically-different strain such as a new variant which can escape the population immunity that has built up? Or you mean that the first wave was controlled before population immunity reached a herd immunity threshold? Even so, if the same strain circulates again, one wouldn't expect a large wave because of the existing immunity from the first wave. Waning immunity in medium-term could also play a role in allowing subsequent epidemics.”*

 We were not, at this early stage in the pandemic, suggesting a further wave from a new variant. Instead, we inferred some heterogeneity in population susceptibility, transmissibility or social interactivity, encapsulated by the phenomenological term, the effective population size at risk of exposure (Peff). This population heterogeneity put a break on virus spread in the first wave but made possible a second wave that moved into less infected sections of the population.  This was unmeasured and not well understood at the time. However, in our subsequent paper (DOI: 10.1126/science.abk0414 ) we were able to explicitly account for this heterogeneity as differences in mobility of lower (high transmission in wave 1) and higher (low transmission in wave 1) socio-economic classes, particularly in the urban setting.

 No change has been made to the manuscript.

*Point  2. "Conclusions - I don't find the Manaus estimate of 75% particularly compelling due to methodological issues in that study. There should be other locations with less extreme first-year serological data?"*

 We agree with the reviewer and revise the section of text referring to the estimate of 75% (see appended). We do already make other comparisons from serosurveys from that period from other settings including Spain, England, United States and Niger. 

 The revised text is 'Our analysis suggests that 30–50% of the urban population were already exposed by the end of September, and that the first wave of the Kenya epidemic peaked in the urban and semi-urban counties during a period of relatively little restrictions or physical distancing. This level of exposure however was not sufficient to prevent a second wave which came shortly after the first (October to December 2020), which we assume to have resulted from heterogeneous spread of the virus, perhaps due to variation in population susceptibility, transmissibility or social interactivity.'

# Peer review: Reviewer response for version 1

**DOI:** 10.21956/wellcomeopenres.18470.r46411

**Published:** 2021-11-01

**Authors:** Amy Wesolowski

**Affiliations:** 1 Department of Epidemiology, Johns Hopkins Bloomberg School of Public Health, Baltimore, MD, USA

In ‘Revealing the extent of the first wave of the COVID-19 pandemic in Kenya based on serological and PCR-test data’ the authors use a range of statistical and mechanistic approaches to investigate the first wave of the pandemic in Kenya using both serological and PCR-test data. Overall, the authors have done an excellent job at integrating appropriate methods with available data sets to provide a useful investigation into transmission patterns in Kenya. Given the dearth of analyses from Sub-Saharan Africa countries, this manuscript is a welcome addition. Overall, I have few comments, the majority of which are minor and outlined below.

 The authors have analyzed transmission patterns across all counties in Kenya, yet only show those for the two most populous (Nairobi and Mombasa) and a country-level result. While this is reasonable for the main text, I believe that adding in results by county to the supplementary information would help the interpretation of the country-level results. Particularly given likely differences in testing and reporting by county, a better understanding of the overall estimates of model predictions and Rt estimates by county would be incredibly valuable. For example, the authors provide the percentage infected by county in Figure 3, however these values are different to interpret in context, particularly without seeing the data and estimates by county.

 In Figure 1 (C,D) the model prediction CIs are incredibly narrow, which seems surprising. Does this occur across the country? Or is this mainly due to the higher quality data in both Nairobi and Mombasa?

 In Figure 2, it would be helpful (perhaps in the supplement or in this main figure) to provide context on when restrictions were lifted in addition to when they were put in place. Further, it is interesting that the IQR for the Rt estimates early in the pandemic seem exceptionally narrow. Additional elaborations on these points (is it likely due to overfitting? Some factors associated with the model fitting? Etc.) should be included. In addition, the Rt estimates for Mombasa in August/September seem substantially different than the rest of the country. Can the authors provide additional context? And do they see similar patterns across the coastal counties during this time?

 The authors do an excellent job appropriately combining different data sets, which is well explained in the supplementary information. Some of these details would be incredibly helpful to move to the main text, in particular additional detail on how the authors treat the serological versus PCR data (and when there were both negative and positive PCR results) and how these two pieces of evidence are integrated.

 Finally, in the supplement the authors use the phrase ‘Chinese epidemic’, but a small point that it may be more appropriate to say ‘epidemic in China’.

Is the work clearly and accurately presented and does it cite the current literature?

Yes

If applicable, is the statistical analysis and its interpretation appropriate?

Yes

Are all the source data underlying the results available to ensure full reproducibility?

Yes

Is the study design appropriate and is the work technically sound?

Yes

Are the conclusions drawn adequately supported by the results?

Yes

Are sufficient details of methods and analysis provided to allow replication by others?

Yes

Reviewer Expertise:

I am an infectious disease epidemiology focused on transmission modeling.

I confirm that I have read this submission and believe that I have an appropriate level of expertise to confirm that it is of an acceptable scientific standard, however I have significant reservations, as outlined above.

# Response

**Published:** 2022-02-03

**Authors:** James Nokes

**Affiliations:** KEMRI-Wellcome Trust Reseach Programme, Kenya

In ‘Revealing the extent of the first wave of the COVID-19 pandemic in Kenya based on serological and PCR-test data’ the authors use a range of statistical and mechanistic approaches to investigate the first wave of the pandemic in Kenya using both serological and PCR-test data. Overall, the authors have done an excellent job at integrating appropriate methods with available data sets to provide a useful investigation into transmission patterns in Kenya. Given the dearth of analyses from Sub-Saharan Africa countries, this manuscript is a welcome addition. Overall, I have few comments, the majority of which are minor and outlined below.

**Thank you for your kind comments on this piece of research.**

 The authors have analyzed transmission patterns across all counties in Kenya, yet only show those for the two most populous (Nairobi and Mombasa) and a country-level result. While this is reasonable for the main text, I believe that adding in results by county to the supplementary information would help the interpretation of the country-level results. Particularly given likely differences in testing and reporting by county, a better understanding of the overall estimates of model predictions and Rt estimates by county would be incredibly valuable. For example, the authors provide the percentage infected by county in Figure 3, however these values are different to interpret in context, particularly without seeing the data and estimates by county.

**This is a very good point. In our original analysis we plotted model prediction intervals against actual data by county as part of model diagnostic. We have now improved our diagnostic visualisations to match the format of the main manuscript plots for Nairobi and Mombasa, and generated county-specific plots for model-based prediction/credible intervals for (i) PCR swab test positives, (ii) population exposure, (iii) deaths, (iv) R(t) against data (where available). All 188 plots (4 x 47 counties) are available in the data and code repository associated with this paper** **https://github.com/ojal/KenyaSerology** **.**

 In Figure 1 (C,D) the model prediction CIs are incredibly narrow, which seems surprising. Does this occur across the country? Or is this mainly due to the higher quality data in both Nairobi and Mombasa?

**This is correct, the reasonably high model certainty about seroprevalence in Nairobi and Mombasa was because of higher data quality in the main cities in Kenya. Other counties had much wider Cis for model predicted seroprevalence, in Figure 3 we attempted to visualise this by candy-striping the county shading for counties with a posterior standard deviation in model prediction of population exposure of greater than 10%; that is the counties where a >10% deviation from the posterior mean estimate of population exposure would not be highly unexpected. We have now added population exposure plots for every county, including credible intervals for the population seropositivity.**

 In Figure 2, it would be helpful (perhaps in the supplement or in this main figure) to provide context on when restrictions were lifted in addition to when they were put in place.

**Over the time scale this paper is concerned with (February – October 2020) there were only two significant relaxations, however, this included lifting the movement restrictions on travel out of Nairobi and Mombasa (6 ^th^ July 2020), and, therefore, was an oversight to not include in Figure 2. We have now revised Figure 2 to include the timing of relaxation of targeted movement restrictions and the reopening of international flight into Kenya (1 ^st^ August 2020). These have been added to a revised Figure 2 and mentioned in the caption of figure 2.**

 Further, it is interesting that the IQR for the Rt estimates early in the pandemic seem exceptionally narrow. Additional elaborations on these points (is it likely due to overfitting? Some factors associated with the model fitting? Etc.) should be included.

**The early tight estimate for Rt reflected (i) fairly tight estimates that Rt ~ 1.1-1.2 in most counties in late April 2020, and (ii) our assumption that for the first 40 days of the simulation (20 ^th^ Feb 2020 – 31 ^st^ March 2020) Rt was proportional to Google data derived estimates of mixing in indoor settings outside the home. In the early stages of the epidemic in Kenya there was very limited testing capacity for detection of SARS-CoV-2 transmission in the community (first positive test result was on 12 ^th^ March 2020). This means that we were forced to make an assumption about the effective relative contact rates, because we could not infer them from epidemiological data. The Google data suggested a fairly uniform 40-45% decrease in mixing in inside settings (e.g., the workplace, etc) outside the home by mid-April 2020, by which time Rt ~ 1.1 - 1.2 in most counties. Because of our modelling assumption, this confidence in Rt in April-May 2020 was translated into confidence in Rt ~ 1.8 – 2.0 during an unobserved epidemic in February 2020.**

**We have added this sentence to the opening paragraph of the *Underlying transmission rates in Mombasa and Nairobi during the first wave* section:**

*“However, we should note that there was very limited PCR testing available in Kenya before April 2020, and our estimates of R(t) pre-April 2020 rely on the assumption that R(t) dropped by ~45% in late March, in parallel to the drop in mobility data (see Methods and supporting data).”*

 In addition, the Rt estimates for Mombasa in August/September seem substantially different than the rest of the country. Can the authors provide additional context? And do they see similar patterns across the coastal counties during this time?

**This is an interesting observation. Upon inspection, other counties in coastal province (Kilifi, Lamu, Tana River and Taita Taveta, but not the Tanzanian border county Kwale) also have a spike in Rt estimates in late August/early September 2020 (see county specific plots). A possibility is that this is connected to the relaxation of movement into Mombasa, however, there is a delay of greater than 4 weeks between that relaxation and the Rt increase.**

 The authors do an excellent job appropriately combining different data sets, which is well explained in the supplementary information. Some of these details would be incredibly helpful to move to the main text, in particular additional detail on how the authors treat the serological versus PCR data (and when there were both negative and positive PCR results) and how these two pieces of evidence are integrated.

**Thank you very much, the aim was not to overwhelm a non-specialist audience, whilst providing full details within the supporting information. We have now added a further paragraph in the Methods section which we hope makes our methodology clearer to the reader.**

**“**The underlying transmission prediction depended only on parameters relevant to infection (e.g. basic pre-measures reproductive ratio etc), however, the statistical modelling of the observation of evidence of these infections varied by type of test and availability of negative PCR test data. Together these form a likelihood function, which integrates the different data sources, since they are all, ultimately, generated by the same underlying infection process. The three statistical models of observation data were:

- **Serological tests**: On each day that serological samples were collected, the log-probability of the observed number of positive tests (ln *f _sero_* (( *ObsS* ^+^) *_n_* |( *S* ^+^) *_n_* , *θ _OM_*)) was assumed to be that of a Beta-Binomial distribution with unbiased sampling of the underlying proportion of serologically detectable people in the county ( ( *S* ^+^) *_n _/N*). The extra dispersion compared to a Binomial sample being due to uncertainty in the underlying sensitivity of the serological assay (see *supporting information *in *supporting data*).
- **PCR swab positive tests when no data on negative PCR tests was available**: Negative PCR swab tests were not available in every county on every day of simulation. When negative swab tests were not available we assumed that the log-probability of the daily observed PCR test positives was from a Negative-binomial distribution:
*μ*_*n*_ *=* *p _test_TR(n)(* *P* ^*+*^ *)*_*n*_
*(Obs* *P* ^*+*^ *)*_*n*_ *∼ NegBin(* *μ ^^^* *=* *μ*_*n*_ *,α ^^^* *= α) *(5)

Where the mean number of daily observed test positives, conditional on the model prediction of PCR-detectable people in the population, is based on sampling a fraction *p*_*test*_ *TR(* *n)* *.* *p*_*test *_was an observation parameter that was jointly inferred during inference, and *TR(n) *was a normalized testing rate based on nationally reported data (see *supporting information *in *supporting data*). *α *was a clustering factor for negative-binomial sampling, jointly inferred with other model parameters.

- **PCR swab positive tests when data on negative PCR tests was available**: When both positive *and* negative PCR test data was available, we assumed that the fraction of positive samples reflected a biased observation of the underlying true fraction of PCR-detectable individuals in the population, e.g. being infected with SARS-CoV-2 could be expected to influence the odds of someone seeking a PCR test. We assumed that the daily detection of PCR test positives could be modelled as samples from a Beta-Binomial distribution with two parameters to infer: 1) The bias of a PCR-detectable individual being PCR tested compared to a PCR-undetectable individual ( *χ*), and, 2) the effective sample size parameter ( *M _PCR_*). *M*_*PCR*_ *→ ∞* recovered a Binomial distribution for the number of positive PCR tests were observed among the tests conducted that day, *M*_*PCR*_ *< ∞ *allowed the model to infer much greater variance in daily proportion of test positives than would be expected from a Binomial distribution. On days where negative swab tests were available, we connect the observable status of epidemic to the data thus,
*p*_*n*_ *=* *χ(* *P* ^*+*^ *)*_*n*_ */ (χ-1)(* *P* ^*+*^ *)*_*n*_** *+ N*
* *
*(Obs* *P* ^*+*^ *)*_*n*_ *∼ BetaBin((* *N* *_s_) ^^^* *=* *N*_*PCR,n*_ *,* *p ^^^* *=* *p*_*n*_ *,* *M ^^^* *=* *M*_*PCR*_ *)*. (6)

Where *N*_*PCR,n *_is the total number of PCR swab samples collected on day *n *and *p*_*n*_ is the proportion of tests performed returning positive expected by the model, accounting for bias in the sampling regime. The bias parameter *χ=1 *recovers an unbiased sample of PCR positives from the underlying population.“

 Finally, in the supplement the authors use the phrase ‘Chinese epidemic’, but a small point that it may be more appropriate to say ‘epidemic in China’.

**Noted, and we have changed our language in the supporting information.**

# Peer review: Reviewer response for version 1

**DOI:** 10.21956/wellcomeopenres.18470.r44753

**Published:** 2021-07-14

**Authors:** Mark Kimathi

**Affiliations:** 1 Department of Mathematics, Machakos University, Machakos, Kenya

The ideas presented in the paper are plausible, resulting to a model, although a simple SEIR model, that is robust and adequately reproduces the observed trajectory of infections. 

 It was captivating to see the authors express the force of infection in terms of the case data time series (which is as a result of "successful" contact between susceptible and infected individuals). This is arguably the only way a compartmental model can reliably reproduce the waves of infections observed in data. Also the notion of, only a proportion of the population is at risk, is a quite realistic.

 There are a few typos, as indicated below:

- On page 3, column 2, in line 10 write as...declining, first in...
- On page 3, column 1, in line 20 write as ...SARS-CoV-2.
- On page 3, column 1, in third paragraph at line 5 write as ...already...

Is the work clearly and accurately presented and does it cite the current literature?

Yes

If applicable, is the statistical analysis and its interpretation appropriate?

Yes

Are all the source data underlying the results available to ensure full reproducibility?

Yes

Is the study design appropriate and is the work technically sound?

Yes

Are the conclusions drawn adequately supported by the results?

Yes

Are sufficient details of methods and analysis provided to allow replication by others?

Yes

Reviewer Expertise:

Mathematical modeling of infectious disease dynamics, fluid flows and other natural phenomena using differential equations.

I confirm that I have read this submission and believe that I have an appropriate level of expertise to confirm that it is of an acceptable scientific standard.

# Response

**Published:** 2022-02-03

**Authors:** James Nokes

**Affiliations:** KEMRI-Wellcome Trust Reseach Programme, Kenya

We thank the reviewer for these comments. The typographical errors noted have been corrected in the revised manuscript.
